# A Psychological Guide to Lower Face Botulinum Toxin Injections: Baseline Emotional Functions of Facial Expressions

**DOI:** 10.1111/jocd.70833

**Published:** 2026-04-16

**Authors:** Alexander G. M. Hopf, Anna Buchheim, Marietta Hopf, Dirk W. Eilert

**Affiliations:** ^1^ Institute of Psychology University of Innsbruck Innsbruck Austria; ^2^ Villa Dahlem Private Practice Berlin Germany

**Keywords:** botulinum toxin, expressive flexibility, facial emotions, facial expressions, facial feedback, resting bitch face

## Abstract

**Background:**

The lower face conveys critical interpersonal signals and is central to how patients are emotionally evaluated by others. As botulinum toxin type A (BoNT‐A) is increasingly used in the perioral and mandibular region, there is a growing need to understand how such procedures may influence both aesthetic outcomes and the dynamics of emotional communication and social perception.

**Aims:**

This narrative review outlines the baseline emotional functions of lower‐face expressions and supports clinicians in integrating emotional and interpersonal considerations into BoNT‐A treatment planning.

**Methods:**

A narrative literature review was conducted using PubMed and Scopus, combining the search terms ‘botulinum toxin’, ‘lower face’, ‘facial expression’, ‘emotion’, ‘mimicry’, and ‘psychology’. Relevant evidence from psychology and neuroscience was synthesized to identify emotional and social signaling functions of key lower‐face Action Units (AUs). Additional insights were integrated through citation tracking and clinical experience.

**Results:**

Lower‐face BoNT‐A targets, such as the depressor anguli oris, platysma, masseter, mentalis, and orbicularis oris, are part of distinct AUs that contribute to emotion expression and interpersonal signaling. Their modulation through BoNT‐A potentially alters cues linked to socially relevant emotions such as sadness and contempt, and social dimensions, including dominance and approachability, thereby affecting how patients are perceived in social contexts.

**Conclusions:**

Understanding the psychological and communicative functions of the lower face helps anticipate how BoNT‐A treatments may influence emotional signaling and social perception. Incorporating these insights into aesthetic consultations supports transparent counseling and strengthens awareness of how aesthetic interventions contribute to nonverbal communication.

## 1 Introduction

The use of botulinum toxin type A (BoNT‐A) in the lower face has gained increasing clinical relevance in recent years, supported by refined injection techniques targeting the depressor anguli oris (DAO), mentalis, platysma, and masseter muscles [1, 2, 3]. While these interventions are primarily performed to improve aesthetic outcomes, such as harmonizing the jawline, correcting downturned oral commissures, or reducing clenching, they inevitably influence the way emotions are communicated and perceived in social interactions. The lower face carries critical interpersonal signals [4]: subtle movements of the lips, chin, and jaw shape impressions of emotions such as sadness, fear and anger, and social dimensions including dominance or approachability, making this region central to the social evaluation of patients [5, 6].

Compared with the upper face, where BoNT‐A has been extensively studied in relation to both inter‐ and intrapersonal outcomes such as mood regulation and baseline emotional functions, psychological research on the lower face remains limited [7, 8, 9]. The recently published *Psychological Guide to* *Upper Face Botulinum Toxin Injections* established a region‐specific framework that mapped the baseline emotional functions of the upper face and highlighted how facial feedback and expressive flexibility are central to understanding aesthetic interventions [10]. Building upon this conceptual foundation, the present review extends the perspective to the lower face, where even subtle changes in perioral and mandibular dynamics can shape how expressions are perceived in everyday social interaction. For clinicians in aesthetic medicine, this underscores the importance of considering not only the technical precision of lower face BoNT‐A injections but also their broader psychological and communicative consequences.

The aim of this narrative review is to integrate psychological and neurobiological perspectives into a coherent understanding of the lower face in aesthetic medicine. By outlining the baseline emotional functions of key facial Action Units (AUs) and the corresponding muscles involved (see Table 1 for details), this paper provides clinicians with a framework to anticipate possible changes in emotional signaling and social perception through lower face BoNT‐A treatments. The goal is to support transparent counseling, enhance treatment planning, and contribute to a deeper awareness of how aesthetic interventions affect nonverbal communication.

**TABLE 1:** Overview of selected lower‐face Action Units (AUs) with their primary muscular basis and corresponding facial movements.

Action Unit (AU)	Muscles involved	Movement
AU9 – Nose Wrinkler	*M. levator labii superioris alaeque nasi*	Wrinkling of the nasal bridge, elevation of the upper lip and nostrils
AU10 – Upper Lip Raiser	*M. levator labii superioris, M. zygomaticus minor*	Raising of the upper lip and widening of the nostrils, exposing the upper teeth or gum line
AU12 – Lip Corner Puller	*M. zygomaticus major*	Upward and diagonal pulling of the lip corners (smiling)
AU14 – Dimpler	*M. buccinator*	Tightening of the mouth corners, forming dimples in the cheek area
AU15 – Lip Corner Depressor	*M. depressor anguli oris*	Downward movement of the lip corners
AU17 – Chin Raiser	*M. mentalis*	Elevation and bulging of the chin; protrusion of the lower lip
AU18 – Lip Pucker	*M. orbicularis* oris	Concentric contraction of the lips, narrowing and forward protrusion of the mouth aperture
AU20 – Lip Stretcher	*M. risorius*, posterior fibers of *M. platysma*	Horizontal stretching of the lips, widening of the mouth
AU23 – Lip Tightener	*M. orbicularis* oris	Tightening of the lips, making the lips appear narrower
AU24 – Lip Presser	*M. orbicularis* oris	Vertical compression of the lips, pressing them firmly together
AU29 – Jaw Thrust	*M. pterygoideus lateralis, M. digastricus*	Forward projection of the mandible, accentuating the chin and broadening the jawline
AU31 – Jaw Clench	*M. masseter,* *M. temporalis*	Vertical compression and mandibular elevation, tightening of the lower face contour

## 2 Materials and Methods

A narrative literature review was conducted to synthesize current knowledge on facial expressions in the lower face and to discuss the potential psychological implications of BoNT‐A treatments in this region. The review aimed to identify the baseline emotional functions of lower‐face Action Units, examine their interpersonal meaning, and summarize key considerations for counseling within aesthetic treatments.

**FIGURE 1:**
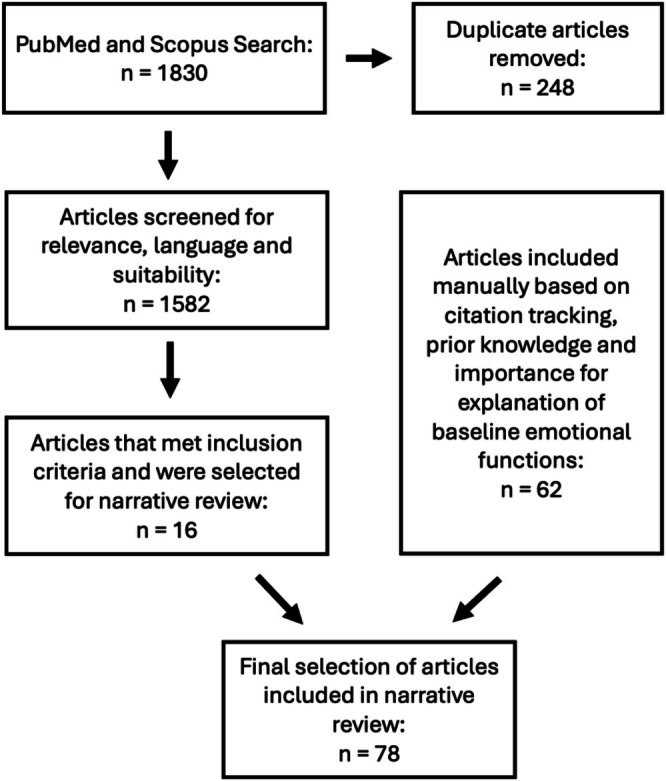
Flowchart depicting search strategy and inclusion criteria. Two evidence streams informed this narrative review: (i) the BoNT‐A evidence stream identified through database screening and (ii) lower face baseline emotional function literature incorporated through citation tracking and prior knowledge.

Searches were carried out in PubMed and Scopus using combinations of the terms “botulinum toxin”, “lower face”, “facial expression”, “emotion”, “mimicry” and “psychology” from database inception to October 2025. Peer‐reviewed articles published in English were included if they addressed emotional, intra‐ or interpersonal aspects of lower‐face expressions or discussed psychological implications of BoNT‐A. Exclusion criteria were non‐English or non‐peer‐reviewed publications, animal or in vitro studies, and BoNT‐A investigations in the upper face or non‐facial regions. Additional literature was identified through citation tracking and through the authors' clinical expertise in aesthetic medicine, psychology, and emotion research. Figure 1 summarizes the identification, screening, and inclusion process for the evidence stream.

Findings were synthesized narratively to highlight the baseline emotional functions of specific lower‐face expressions, explore the potential impact of BoNT‐A modulations on social perception and provide an outlook on their relevance for patient communication, counseling and future empirical studies.

The description of baseline emotional functions of specific lower‐face Action Units was based on a previous framework [6], which offers a comprehensive reference for linking AUs to emotional signaling.

For manuscript preparation, ChatGPT‐5 was used exclusively for language refinement. All scientific content, clinical interpretations, and conceptual frameworks were developed by the authors.

## 3 Results and Discussion

### 3.1 The Perioral Region

#### 3.1.1 Baseline Emotional Functions of Perioral Facial Expressions

The perioral region encompasses a wide range of expressive movements that shape both emotional communication and social perception. Key Action Units in this area include AU9 (Nose Wrinkler), AU10 (Upper Lip Raiser), AU12 (Lip Corner Puller), AU14 (Dimpler), AU18 (Lip Pucker), as well as AU23 (Lip Tightener) and AU24 (Lip Presser). These movements interact dynamically to convey emotions such as interest, sexual desire, disgust, or contempt, as well as social displays, including a polite smile [6].

##### 3.1.1.1 AU9–Nose Wrinkler and AU10–Upper Lip Raiser

**FIGURE 2:**
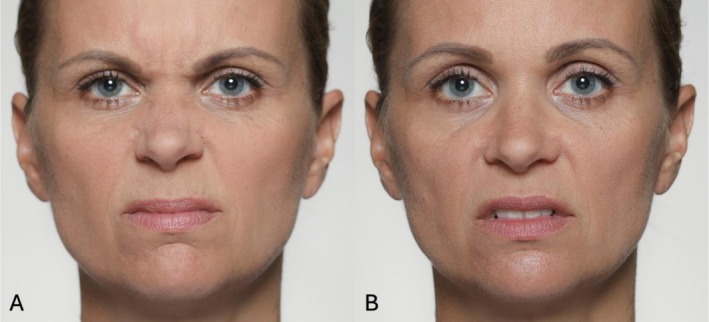
Illustrative photographic depiction of AU9 (A) and AU10 (B). AU9 involves the levator labii superioris alaeque nasi, producing a pronounced wrinkling of the nasal bridge. In contrast, AU10 primarily activates the levator labii superioris and the zygomaticus minor, raising the upper lip without engaging the nasal area.

- Disgust: Wrinkling of the nose and raising of the upper lip are considered core movements of disgust expressions. Whilst AU9 typically signals disgust, which is triggered by unpleasant odors or tastes, upper lip raising (AU10) is more indicative of moral disgust or rejection of socially inappropriate behavior [11, 12]. For a detailed differentiation between AU9 and AU10, see Figure 2.
- Skepticism: A subtle form of AU9 can also indicate inner objection or doubt in the form of skepticism [13].
- Contempt: Unilateral expression of AU10 can appear in contemptuous or hostile expressions [14].
- Pain: AU9 and AU10 both belong to the core movements of pain expressions, frequently observed in both acute and chronic pain contexts [15].
- Sexual desire: Bilateral or unilateral upper lip raising may also signal sexual interest, particularly when combined with body‐scanning gaze cues. Bilateral AU10 may occur just before orgasm, indicating anticipated sexual satisfaction, while unilateral AU10 can function as a status‐driven signal of sexual conquest [16].

##### 3.1.1.2 AU12–Lip Corner Puller and AU14–Dimpler

- Joy: Elevation of the lip corners in AU12, when part of a Duchenne smile, is a primary indicator of joy and social affiliation [17].
- Social vs. Duchenne (authentic) smile: The differentiation between social smiles and Duchenne smiles can be identified by AU6 (Lid Compressor) in the lateral canthal region [18].
- Affiliative emotions: Smiling is a core element of several other emotions, including embarrassment, pride, and love [6].
- Superiority: A unilateral smile often conveys superiority and the activation of dominance or assertion motives [19].
- Contempt: A unilateral AU14 differs from a smile by pulling the lip corner laterally rather than upward, due to the action of the buccinator muscle. When combined with a slight head tilt backward or a brief break in eye contact, it may signal contempt [20, 21].

**FIGURE 3:**
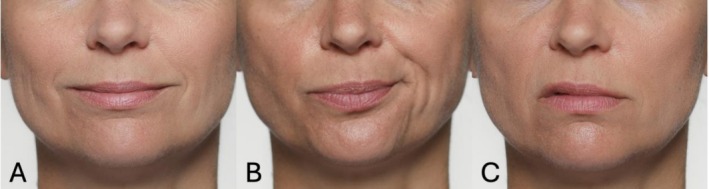
Illustrative photographic depiction of AU12 (A), unilateral AU14 (B), and unilateral AU10 (C). AU12 (Lip Corner Puller) results from contraction of the zygomaticus major, elevating the lip corners upward and diagonally. AU14 (Dimpler) involves activation of the buccinator muscle, pulling the lip corners laterally and creating a slight indentation or dimple in the cheek area. Unilateral AU10 (Upper Lip Raiser) is produced by the levator labii superioris, lifting the upper lip vertically and exposing the upper teeth or gum line on one side.

For a detailed differentiation of AU12, AU14, and a unilateral AU10, see Figure 3.

##### 3.1.1.3 AU18–Lip Pucker

- Interest and curiosity: Puckering the lips may signal attentional engagement and exploratory interest [22].
- Flirtation and sexual interest: Lip puckering in the presence of a potential romantic partner can indicate sexual interest, particularly when combined with body‐scanning gaze behavior [23].
- Cognitive objection: During listening, AU18 may mark inner disagreement or objection to the speaker's statement [6, 24].
- Thoughtfulness: Lip puckering can also indicate inner deliberation or weighing of options [13].

**FIGURE 4:**
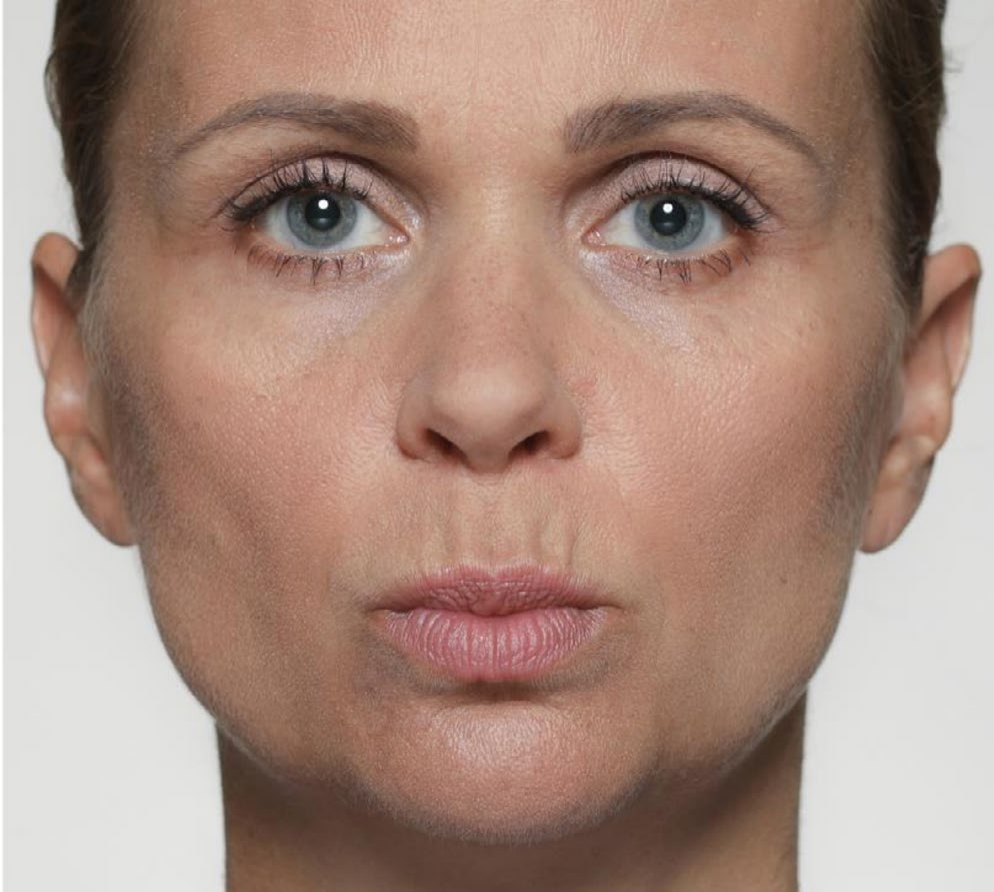
Illustrative photographic depiction of AU18 (Lip Pucker). This movement results from concentric contraction of the orbicularis oris muscle, drawing the lips forward into a rounded shape.

See Figure 4 for an example of AU18.

##### 3.1.1.4 AU23–Lip Tightener and AU24–Lip Presser

**FIGURE 5:**
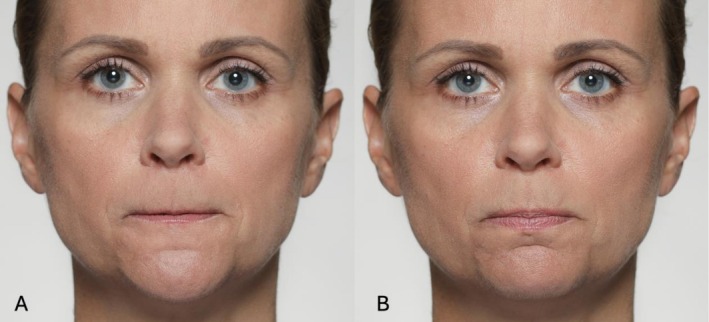
Illustrative photographic depiction of AU23 (A) and AU24 (B). Both movements involve activation of the orbicularis oris muscle complex but differ in direction and intensity. AU23 (Lip Tightener) tightens the lips, making them appear narrower. AU24 (Lip Presser) adds vertical compression, pressing the lips firmly together.

- Anger: Tightening the lips is a reliable signal of anger, conveying readiness for confrontation. Lip pressing may indicate mild (‘cold’) or controlled (‘hot’) anger, though it is not a reliable cue when shown in isolation [25]. Figure 5 shows the difference between AU23 (Lip Tightener) and AU24 (Lip Presser).
- Cognitive load: When appearing more frequently than baseline, lip pressing can be a sign of elevated cognitive load, such as during intense thinking or deception [26, 27, 28].
- Self‐control: Lip pressing occurs when individuals attempt to suppress emotional expressions, functioning as a nonverbal marker of emotional self‐regulation [26, 29]
- Embarrassment: In combination with gaze aversion, lip pressing can signal embarrassment, especially when used to suppress a smile [30].
- Compassion: When displayed while listening, combined with nodding and inner brow raising, lip pressing can be interpreted as a cue of compassion [31].

#### 3.1.2 Interpersonal Effects

Activation of the levator labii superioris and levator labii superioris alaeque nasi, expressed as upper lip elevation and nose wrinkling (AU10 and AU9), is a strong rejection cue that signals disgust, which acts as a social distancing cue [11].

Even in the absence of overt emotional expressions, subtle tonic activations or structural facial features in the perioral and mandibular regions can influence how a neutral face is socially perceived. The phenomenon commonly referred to as *Resting Bitch Face* (RBF) illustrates how neutral facial configurations can nevertheless convey negative affective qualities such as rejection, contempt, or disapproval. Empirical findings demonstrated that *perceived resting negative emotion*–a composite index of anger, disgust, fear, sadness, and low happiness–systematically predicts decreased attractiveness and increased perceived threat, particularly for female participants [32]. These results emphasize that minimal, trait‐like facial configurations or habitual muscle tonus in specific lower‐face Action Units may bias observers toward impressions of emotional negativity and interpersonal distance.

From a psychophysiological perspective, such impressions can arise both from subtle, tonic co‐activations and from static facial structures that merely resemble the morphology of expressive Action Units. Elevation of the upper lip and wrinkling of the nasal bridge (AU10, AU9) communicate disgust and social distancing. Lateral tightening of the lip corners (AU14) or horizontal compression of the lips (AU23) convey dominance, contempt, or restrained anger. When these patterns are mimicked by baseline muscle tone–such as slightly raised upper lips or laterally tightened mouth corners–they can simulate the visual appearance of disgust or contempt, even in the absence of actual muscle activation. This resemblance reinforces the perception of aloofness or unapproachability often attributed to RBF [32]. Clinically, awareness of these structural or tonic cues is essential, as BoNT‐A interventions in these regions may modulate not only dynamic expressions but also baseline impressions in social interaction.

In contrast, contraction of the zygomaticus major (AU12) in smiling communicates openness and affiliation. Smiling increases perceived attractiveness, intelligence, and self‐confidence [33, 34, 35] and makes others more likely to approach [36]. It also influences intrapersonal states, leading to friendlier evaluations of others and greater perceived energy during physical activity [37, 38]. However, smiling is context‐dependent; in a stressful social situation, it may seem inappropriate to others, leading them to perceive someone as less likable [39]. The presence of AU6 remains central for distinguishing social smiles from genuine joy.

Tension in the orbicularis oris (AU23 and AU24) conveys anger, assertiveness, and dominance, often reducing approachability. This must be distinguished from lip sucking (AU28), which represents a self‐soothing gesture under stress and can also appear in states of nervousness [40]. Especially in the perioral region, structural and habitual lip features can influence social perception. Thin or naturally tense lips, for instance, can create unintended impressions of anger by simulating an AU23/24 appearance.

#### 3.1.3 Neuropsychological Insights

Experiencing disgust or contempt reduces neuronal activity in brain regions involved in empathy processing [41]. However, from a neuropsychological perspective, lower‐face movements are comparatively easier to regulate voluntarily than those of the upper face, making ‘masking’ of emotions easier. A study on deception showed that participants attempting to suppress facial expressions could reduce smiling or brow contraction but not eliminate them completely. Interestingly, suppression was more successful in the lower face than in the upper [42]. This difference is rooted in cortical representation: in the motor cortex, lips occupy a disproportionately large area, allowing fine‐grained voluntary control, while the brow region has only limited representation and is thus harder to inhibit [43].

#### 3.1.4 Key Considerations for Counseling

The perioral area encompasses a variety of different movements that shape interpersonal communication. For clinicians, this means that hyperactivity or an AU‐like baseline appearance in the perioral region can unintentionally generate impressions of rejection, tension, or unapproachability, even in neutral states. Key considerations in this area are summarized in the Appendix Table A1.

BoNT‐A offers targeted options to soften such signals. Treatment of the levator labii superioris alaeque nasi can have a profound effect on muscular dynamics and facial expressions [44]. Reducing its activity can diminish exaggerated disgust expressions and improve the impression of social openness. Reducing these muscles is typically a part of the treatment of a ‘gummy smile’ [45]. Subtle weakening of the orbicularis oris, as in the “lip flip”, not only increases vermilion show but also reduces the appearance of tightly held lips associated with AU23 and AU24 [46]. Beyond BoNT‐A, dermal fillers play an essential role in lip augmentation [47]. Thin lips have been linked to perceptions of anger or aloofness [48]. Restoring volume and contour can counteract such unintended impressions, enhancing both facial harmony and social approachability.

However, perioral interventions require nuance. Lip pressing is not only a marker of anger but can also signal thoughtfulness or cognitive control. Over‐suppressing such movements risks diminishing authentic nonverbal cues that play an important role in empathy and intrapersonal emotion regulation.

### 3.2 The Mandibular Region

#### 3.2.1 Baseline Emotional Functions of Mandibular Facial Expressions

The mandibular region comprises several Action Units that contribute to expressions of sadness, compassion, fear, anger, and stress. Key movements include AU15 (Lip Corner Depressor), AU17 (Chin Raiser), AU20 (Lip Stretcher), AU29 (Jaw Thrust), and AU31 (Jaw Clench). These AUs interact to convey emotional states related to both vulnerability and confrontation.

##### 3.2.1.1 AU15–Lip Corner Depressor

- Sadness: Downward pulling of the lip corners is a prototypical cue for controlled or low‐intensity sadness, signaling disappointment or dejection [5, 49].
- Affiliation and harmony: The movement is associated with the activation of affiliative motives, emphasizing connectedness and social attunement [6].

##### 3.2.1.2 AU17–Chin Raiser

- Sadness: Elevating the chin bulge is a prototypical sadness cue. A trembling chin is a reliable precursor of crying, especially in children, marking the onset of intense sadness [31, 50, 51].
- Compassion: When paired with forward head tilt or affiliative gestures, AU17 can signal compassion [31].
- Thoughtfulness: AU17 may also appear during cognitive reflection, often combined with lip pressing [6].
- Offense and defiance: When co‐activated with anger signals, AU17 indicates insult, defiance, or stubborn resistance [52].

**FIGURE 6:**
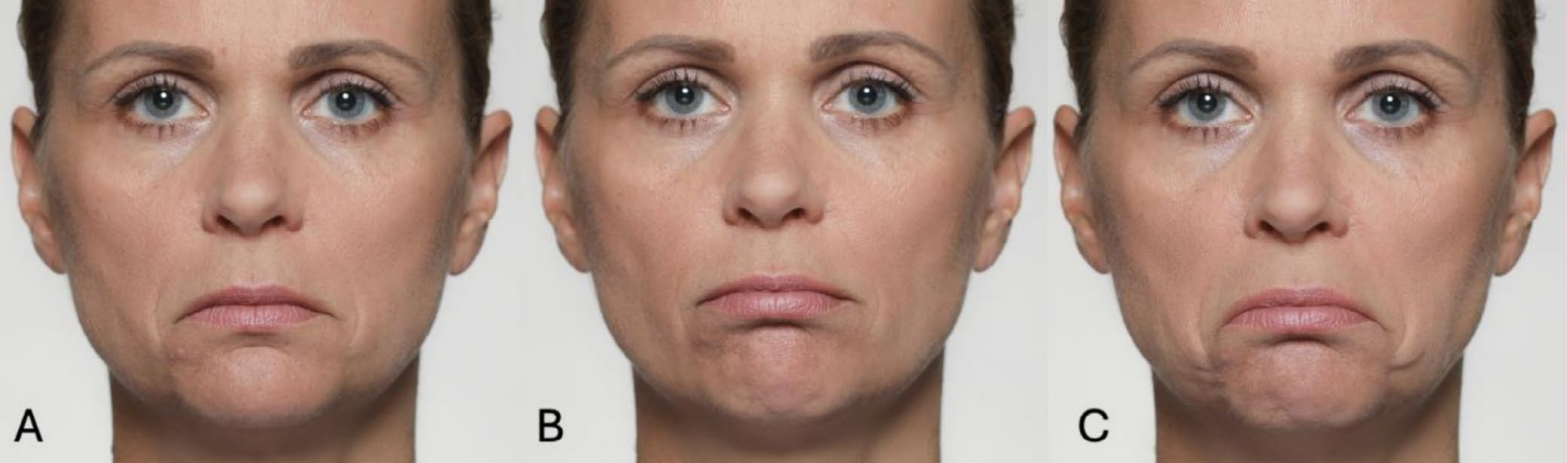
Illustrative photographic depiction of AU15 (A), AU17 (B), and combined AU15 + AU17 activation (C). AU15 (Lip Corner Depressor) involves contraction of the depressor anguli oris, pulling the lip corners downward. AU17 (Chin Raiser) results from activation of the mentalis muscle, producing a vertical elevation and bulging of the chin. The combined activation of AU15 and AU17 leads to simultaneous downward movement of the lip corners and upward projection of the chin, leading to the so‐called ‘facial shrug’–the facial equivalent of the culturally universal shrug gesture.

See Figure 6 for a differentiation of AU15, AU17, and a combination of AU15 + 17.

##### 3.2.1.3 AU20–Lip Stretcher

- Fear: Horizontal stretching of the lips is a core feature of fear expressions, especially when combined with raised upper eyelids [5, 53].
- Pain: Also observed in pain displays, contributing to the widening of the mouth aperture [54].

**FIGURE 7:**
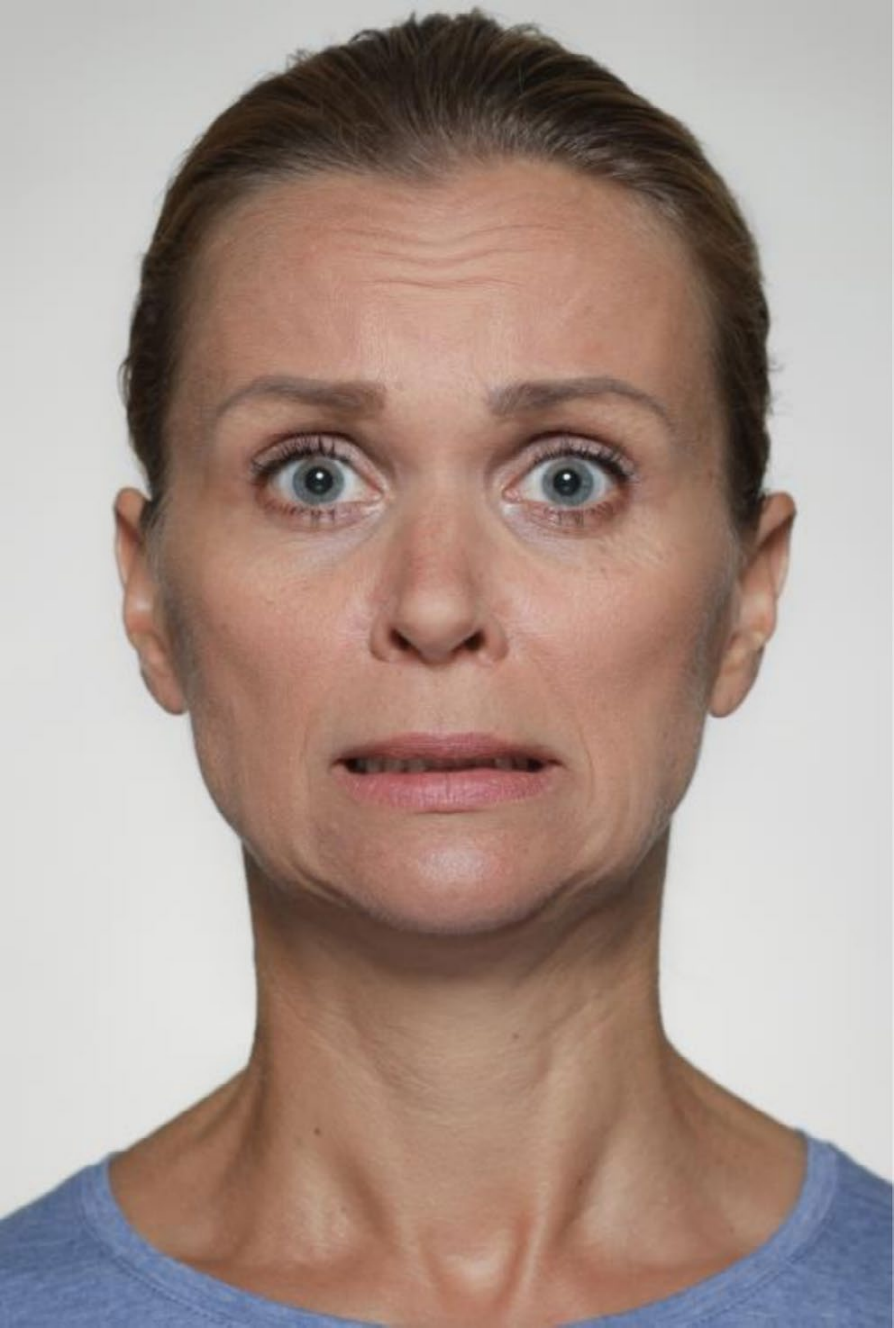
Illustrative photographic depiction of AU20 (Lip Stretcher). This movement is produced by bilateral activation of the risorius and posterior fibers of the platysma, horizontally stretching the lips and widening the mouth.

See Figure 7 for an example of AU20.

##### 3.2.1.4 AU29–Jaw Thrust and AU31–Jaw Clench

**FIGURE 8:**
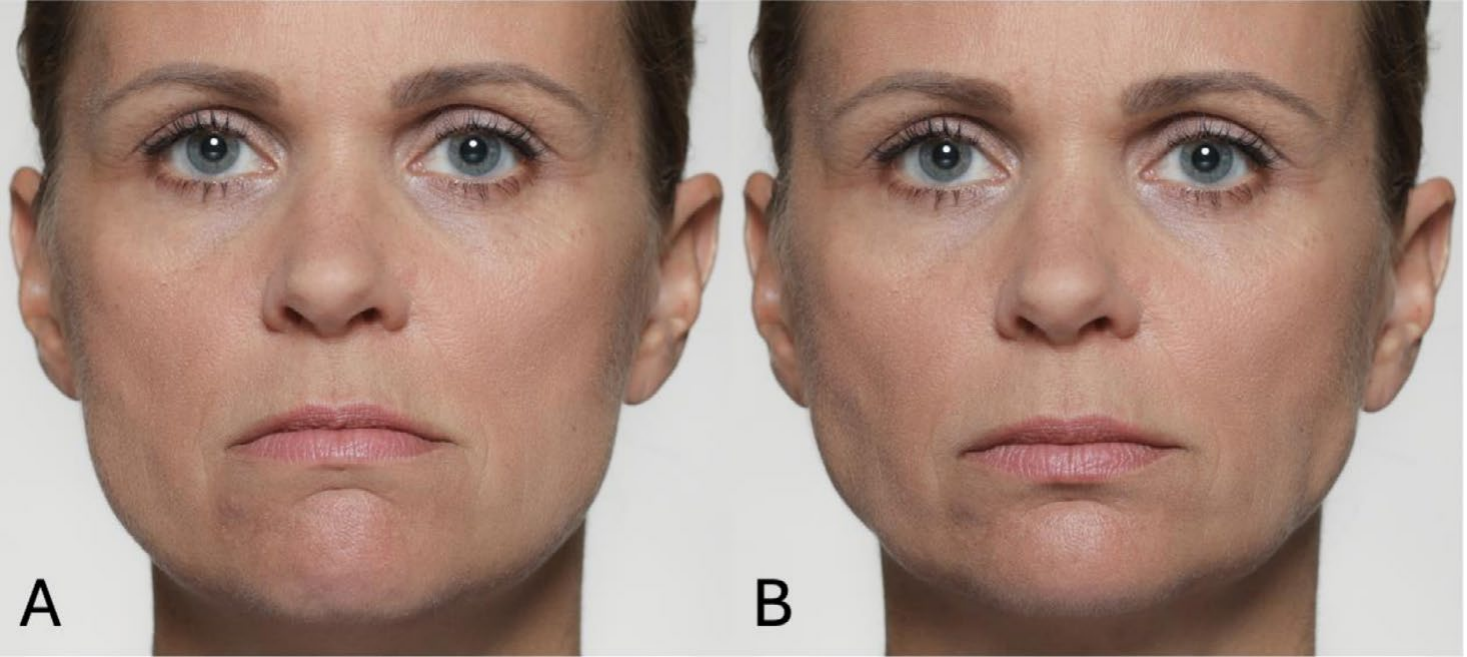
Illustrative photographic depiction of AU29 (A) and AU31 (B). AU29 (Jaw Thrust) involves contraction of the pterygoid and digastric muscles, producing a forward projection of the mandible. AU31 (Jaw Clench) is characterized by activation of the masseter and temporalis muscles, generating vertical compression and mandibular elevation. This results in a tightened lower facial contour and pronounced muscular tension along the jawline.

- Anger and conflict: Both jaw thrusting and jaw clenching can signal anger, particularly when co‐occurring with other agonistic AUs such as AU4 (Brow Lowerer) or AU23 (Lip Tightener). AU29 contributes through forward projection, which accentuates readiness for confrontation, while AU31 reflects muscular tension that accompanies escalating anger [5, 50]. See Figure 8 for a differentiation of AU29 and AU31.
- Dominance: Jaw thrust broadens the jawline and accentuates the chin, enhancing perceived dominance [55].
- Stress and cognitive load: Clenched jaws are a hallmark of emotional stress and are also observed during high cognitive effort [56, 57, 58].
- Physical strain: Both movements appear under muscular exertion, reflecting effort and tension [59].

#### 3.2.2 Interpersonal Effects

Expressions in the mandibular region are decisive for how patients are socially perceived, often tipping impressions toward vulnerability or dominance. Contraction of the DAO pulls the mouth corners downward (AU15), reliably communicating sadness. In contrast, activation of the risorius and platysma stretches the lips laterally (AU20), a hallmark of fear and potentially pain. Crucially, both AU20 and AU15 have been shown to reduce perceived assertiveness and lower social status, whilst AU15 also signals interpersonal warmth [60, 61].

The mentalis muscle (AU17) intensifies these impressions: a trembling chin is a precursor of crying and signals emotional fragility, while in combination with anger cues, it can flip to defiance [52]. When DAO and mentalis contract together, the result is the so‐called “facial shrug”, typically interpreted as disbelief or denial [5, 62].

By contrast, the masseter and temporalis muscles contribute to expressions of strength. Forward projection of the jaw (AU29) or forceful clenching (AU31) accentuates dominance, anger, and assertiveness, but also reveals stress or muscular strain when habitual [55, 63]. These movements stand in sharp opposition to the affiliative or vulnerable cues of DAO, mentalis, and platysma activity.

#### 3.2.3 Neuropsychological Insights

Empirical evidence underscores that smiling itself engages potent facial feedback mechanisms relevant for the lower face. Soussignon [64] demonstrated that genuine (Duchenne) smiles–combining zygomatic and periorbital activation–intensify pleasant affect, suggesting that feedback from lower‐face musculature contributes to emotional experience [64]. The Many Smiles Collaboration confirmed this link in a global replication, showing that voluntary smiling increased happiness, whereas artificially induced mouth positions (as in the pen‐in‐mouth task) did not [65]. These findings indicate that lower face movements can influence the modulation of emotions. However, to the best of our knowledge, the specific impact of BoNT‐A treatments on these facial feedback mechanisms in the lower face has not yet been systematically investigated. Specifically, BoNT‐A treatment of the depressor anguli oris muscle indirectly elevates the corners of the mouth, thereby facilitating a facial configuration associated with increased approach‐motivation and pleasant affect. However, to this date, no controlled studies have specifically tested whether mouth‐corner lifting via reduction of depressor anguli oris activity yields a mood‐enhancing effect, and thus this hypothesis awaits empirical validation in future research.

Furthermore, mandibular movements illustrate the interplay between trait and state signals. At rest, structural or habitual cues–such as downturned lip corners or a tense jawline–can bias observers toward perceiving sadness or anger, even in the absence of emotion [66]. By contrast, microexpressions in this region, such as a fleeting AU15 or a rapid jaw clench (AU31), reflect momentary emotional states that are difficult to suppress. For clinicians, this distinction is crucial: while transient activations may briefly modulate expression, it is the stable, trait‐like configuration of the lower face that predominantly shapes social attribution and thus determines how approachable or dominant a patient appears in everyday interactions.

#### 3.2.4 Key Considerations for Counseling

The mandibular region is central to the expression of sadness, vulnerability, dominance, and fear, and its modulation through botulinum toxin has direct implications for how patients are socially perceived. Treating the depressor anguli oris (AU15) can effectively lift drooping mouth corners [67], reducing impressions of sadness or resignation that otherwise dominate a neutral expression, thereby potentially elevating mood. In the mentalis (AU17), BoNT‐A can soften chin dimpling and trembling, thereby lowering the perception of vulnerability, though overtreatment risks blunting subtle affiliative cues of compassion.

The masseter is both an aesthetic and functional target [68, 69]: reducing clenching alleviates stress‐related tension and bruxism, while also softening the appearance of aggressiveness or dominance. At the same time, clinicians must consider that such treatments may diminish the expression of strength and determination.

Particularly relevant for the emotional reading of the lower face is the platysma. Its activation underlies many fear‐related expressions, stretching the lips laterally and lowering the perceived social status. Relaxing the platysma with BoNT‐A has been shown to improve lower facial contour [70], potentially reducing exaggerated fear or submissiveness, creating a calmer, more composed appearance. Key considerations in this area are summarized in the Appendix Table A2.

## 4 Conclusion

In the past years, a considerable effort has been undertaken to investigate optimal treatment strategies with BoNT‐A for the lower face [71]. At the same time, a nuanced understanding of baseline functions and the interpersonal meaning of lower‐face expressions is essential for patient counseling [72]. Integrating these insights enables clinicians to align aesthetic outcomes with patients' desired social perception during interpersonal communication.

Compared with the upper face, where the facial feedback hypothesis has been repeatedly linked to intrapersonal effects such as mood modulation, research on the actual effect of BoNT‐A treatments in the lower face on intrapersonal experience is missing [73, 74]. Most available evidence highlights the interpersonal dimension–how mandibular and perioral signals shape approachability, dominance, or vulnerability–while systematic data on their influence on the intrapersonal emotional states of the patient is scarce. Addressing this gap is crucial for a balanced understanding of aesthetic interventions, such as BoNT‐A.

In addition, future research should examine how BoNT‐A interventions may influence intrapersonal experience and interpersonal communication in dyadic contexts, particularly regarding attachment‐related processes. Previous studies have shown that individual attachment representations modulate both emotion regulation and expression, as well as intra‐ and interpersonal emotion perception [75, 76]. These domains correspond to the core functions of emotion processing (C‐FEP) [77] empathy, impathy, emotion regulation, and expression–which are also likely to be affected by BoNT‐A interventions. Investigating these interactions may therefore help bridge neurobiological mechanisms of facial feedback with the psychological dynamics of emotional communication.

At the same time, the lower face illustrates that the impact of aesthetic treatments extends beyond botulinum toxin. Lip augmentation with hyaluronic acid, for example, not only restores contour but can counteract impressions of anger or aloofness associated with thin lips, thereby reshaping affiliative signals [78]. Such findings call for future research that investigates both neurotoxin and filler interventions in terms of their psychological and communicative consequences.

To translate these insights into clinical practice, future progress will depend on developing tools that help patients express how they wish to be perceived by others, and on clinicians being able to map these interpersonal effects to concrete treatment strategies. Bridging this gap between patient goals and therapeutic options will elevate routine aesthetic practice beyond cosmetic improvement, positioning it as a discipline that actively shapes social perception and emotional well‐being.

## Author Contributions

Alexander G.M. Hopf Anna Buchheim, Marietta Hopf, and Dirk W. Eilert were all involved in the conceptualization and planning of the manuscript. Alexander G.M. Hopf, drafted the initial manuscript, and Anna Buchheim, Marietta Hopf, and Dirk W. Eilert revised and contributed to the individual sections. All authors approved the final version of the manuscript.

## Funding

The authors have nothing to report.

## Disclosure

A. G. M. H. is Director of International Professional Education at Evolus Inc., Newport Beach, California, USA.

## Ethics Statement

The authors confirm that the ethical policies of the journal, as noted on the journal’s author guidelines page, have been adhered to. No ethical approval was required as this is a review article with no original research data. Written informed consent was provided by all identifiable people in the photographs.

## Conflicts of Interest

A. G. M. H. is an employee of Evolus Inc., Newport Beach, California, USA. MH is a speaker for Croma Pharma, Leobendorf, Austria. The other authors declare no conflicts of interest.

## Data Availability

Data sharing not applicable to this article as no datasets were generated or analysed during the current study.

## Appendix A

**TABLE A1:** Key considerations for the perioral region: Baseline emotional functions and potential changes with reduced activity through BoNT‐A.

Action Unit (AU)	Baseline emotional functions	Potential changes with reduced activity (BoNT‐A)
AU9–Nose Wrinkler	Core movement of disgust expression; signals rejection and social distancing; also observed in pain expressions.	Reducing activity in this area can diminish exaggerated disgust expressions and improve the impression of social openness.
AU10 – Upper Lip Raiser	Indicates moral disgust; unilateral activation can signal contempt or hostility.	Reduced activity may soften excessive upper‐lip elevation and lower the appearance of rejection cues.
AU12 – Lip Corner Puller	Core movement of joy and social affiliation; central to smiling and the Duchenne smile.	Reduction of antagonistic downward pull (AU15) may enhance the ability to smile and increase the visibility of joy‐related cues.
AU14 – Dimpler	Tightening the mouth corners; signals contempt or social dominance.	Not a typical target for a specific BoNT‐A treatment.

**TABLE A2:** Key considerations for the mandibular region: Baseline emotional functions and potential changes with reduced activity through BoNT‐A.

Action Unit (AU)	Baseline emotional functions	Potential changes with reduced activity (BoNT‐A)
AU15 – Lip Corner Depressor	Communicates sadness, disappointment, or dejection.	Reduction can lift drooping mouth corners, decreasing sadness cues and enhancing the ability to smile.
AU17 – Chin Raiser	Indicates sadness, compassion, or defiance depending on context.	Reduction softens dimpling and trembling, lowering perceived vulnerability; overtreatment may blunt affiliative cues.
AU20 – Lip Stretcher	Expresses fear and submission through lateral lip stretching.	Relaxation can reduce exaggerated fear or submissiveness and create a calmer, more composed appearance.
AU29 – Jaw Thrust	Signals anger, confrontation, and dominance.	Not a typical target for a specific BoNT‐A treatment
AU31 – Jaw Clench	Associated with anger, stress, and muscular strain.	Relaxation can reduce stress‐related tension and soften an appearance of aggressiveness or dominance.
